# Deep Learning-Based Multiclass Brain Tissue Segmentation in Fetal MRIs

**DOI:** 10.3390/s23020655

**Published:** 2023-01-06

**Authors:** Xiaona Huang, Yang Liu, Yuhan Li, Keying Qi, Ang Gao, Bowen Zheng, Dong Liang, Xiaojing Long

**Affiliations:** 1 Research Center for Medical AI, Shenzhen Institute of Advanced Technology, Chinese Academy of Sciences, Shenzhen 518055, China; 2 Department of Artificial Intelligence, University of Chinese Academy of Sciences, Beijing 100049, China; 3 Shenzhen Maternity and Child Healthcare Hospital, Shenzhen 518027, China

**Keywords:** deep learning, medical image segmentation, fetal MRI, convolution

## Abstract

Fetal brain tissue segmentation is essential for quantifying the presence of congenital disorders in the developing fetus. Manual segmentation of fetal brain tissue is cumbersome and time-consuming, so using an automatic segmentation method can greatly simplify the process. In addition, the fetal brain undergoes a variety of changes throughout pregnancy, such as increased brain volume, neuronal migration, and synaptogenesis. In this case, the contrast between tissues, especially between gray matter and white matter, constantly changes throughout pregnancy, increasing the complexity and difficulty of our segmentation. To reduce the burden of manual refinement of segmentation, we proposed a new deep learning-based segmentation method. Our approach utilized a novel attentional structural block, the contextual transformer block (CoT-Block), which was applied in the backbone network model of the encoder–decoder to guide the learning of dynamic attentional matrices and enhance image feature extraction. Additionally, in the last layer of the decoder, we introduced a hybrid dilated convolution module, which can expand the receptive field and retain detailed spatial information, effectively extracting the global contextual information in fetal brain MRI. We quantitatively evaluated our method according to several performance measures: dice, precision, sensitivity, and specificity. In 80 fetal brain MRI scans with gestational ages ranging from 20 to 35 weeks, we obtained an average Dice similarity coefficient (DSC) of 83.79%, an average Volume Similarity (VS) of 84.84%, and an average Hausdorff95 Distance (HD95) of 35.66 mm. We also used several advanced deep learning segmentation models for comparison under equivalent conditions, and the results showed that our method was superior to other methods and exhibited an excellent segmentation performance.

## 1. Introduction

Congenital diseases are one of the leading causes of neonatal death worldwide. To comprehensively understand the neurodevelopment of normal fetuses and fetuses with congenital disorders and realize early detection and treatment of congenital disorders, prenatal maternal and infant health screening and quantitative analyses of the developing human fetal brain are essential. Ultrasonography is an important tool for prenatal identification and screening of fetal malformations, but detecting abnormal development of some tissues, such as the cerebellar vermis, is often limited due to the influence of fetal position and cranial acoustic attenuation on ultrasonography. In recent years, fetal magnetic resonance imaging (MRI) has become an important auxiliary tool that can provide more information on fetal development in the case of unclear ultrasound images to improve the possibility and accuracy of congenital disease diagnoses. Evidence has demonstrated that fetal MRI, especially brain MRI, can be a useful diagnostic tool for many congenital disorders, such as spina bifida, intrauterine growth retardation, congenital heart disease, anencephaly, corpus callosum anomalies, and more [1,2].

Important neurodevelopmental changes occur in the last trimester of pregnancy, between 30 and 40 weeks of gestation, and include volume growth, myelination, and cortical gyration. Fetal MR images allow clinicians to monitor fetal brain development in utero at an early developmental stage and detect brain abnormalities early. Fetal brain MRI analysis typically begins by segmenting the brain into different tissue categories, followed by volumetric and morphological analyses. This is because many congenital diseases cause subtle changes in these tissues [3]. The incidence rate of congenital heart disease (CHD) accounts for approximately 5–8% of all live births every year and is the most common congenital malformation and an important cause of neonatal death. Licht et al. [4] examined 25 neonates with CHD using MRI and found that 53% had brain damage, including microcephaly (24%), incomplete fontanel closure (16%), and periventricular leukomalacia (PVL) (28%). White matter damage was characteristic of brain damage in premature infants. Hypoplastic left heart syndrome (HLHS) is one of the most severe forms of congenital heart disease (CHD). The volume of cortical gray matter, white matter, and subcortical gray matter of HLHS fetus in the third trimester of pregnancy gradually decreases. In addition to white matter (WM), gray matter (GM), and cerebrospinal fluid (CSF), other tissues, such as the cerebellum (CB) and brainstem (BS), are equally important for understanding and predicting healthy or abnormal brain development in preterm infants of similar gestational age. The cerebellum is particularly clinically important because it is one of the fastest-growing brain regions in the last trimester of pregnancy. The Dandy–Walker malformation, for example, corresponds to changes in brain tissue with hypoplasia or absence of the cerebellar earthworms, hypoplasia of the cerebellar hemispheres, and enlargement of the posterior cranial fossa and fourth ventricle. The enlargement of the posterior fossa results in an elevation of the torcula that is nicely demonstrated on the sagittal images, as is associated hypoplasia of the brain stem. A prenatal ultrasound can show some of the features of Dandy–Walker malformation, but is limited in its ability to assess vermian hypoplasia. Therefore, the fetal MRI allows for a better evaluation of these anomalies. In contrast, spina bifida is associated with the brainstem and ventricle, and research [5] has shown that in fetuses with open spina bifida (OSB), the brainstem diameter is higher compared to normal fetuses, which may be the result of caudal displacement of the brainstem and compression of the fourth ventricle-large magna complex within the confined space between the sphenoid and occipital bones. T. GHI et al. [6] studied 66 fetuses with isolated spina bifida, with a mean gestational age at diagnosis of 21 weeks (range 16–34 weeks) and 56 cases diagnosed before 24 weeks. A total of 59 cases chose to terminate the pregnancy and 7 fetuses were delivered alive and survived. Fifty-seven cases representing the study group were followed up in detail. About 53 (93.0%) of the postnatal defects were classified as open and 4 (7.0%) cases had closed defects (three lipohypophysis, one meningocele). At mid-gestation, ventricular enlargement was present in 34/53 cases (64.2%). Therefore, it can be seen that the detection of morphological changes is of great value for congenital diagnosis.

Fetal MRI is a challenging imaging modality because the fetus is not sedated and tends to move freely, causing some artifacts. The imaging technique uses ultrafast MRI sequences, such as T2-weighted single-shot fast spin echo (ssFSE), which allows very rapid acquisition of low-resolution images, and super-resolution reconstruction algorithms can then be applied to merge several low-resolution images into a single high-resolution volume. Traditionally, physicians manually segmented these images into several tissues. However, this manual segmentation is tedious and time-consuming because of the small fetal brain volume and the partial volume effects of different tissue classes make manual segmentation error-prone and must be performed by highly specialized clinicians. Due to the difficulty of the segmentation task, many automated methods have been attempted to detect and segment brain tissues to aid in diagnosis.

Automated fetal brain tissue segmentation is challenging because the spontaneous movements of the fetus and the mother during the scanning process cause some artifacts, such as uneven intensity, making it difficult to distinguish between tissue types, as well as the complex shapes of different tissues, which can also pose some challenges for segmentation.

Atlas-based segmentation techniques rely on an image registration process that looks for voxel-to-voxel alignment of the two images. One image is an atlas from which a labeled map of the structure of interest can be obtained, and the other is an image of the target subject to be segmented. For the automatic segmentation of fetal brain tissue in reconstructed MR volumes, Habas et al. [7] proposed an atlas-based approach for automatic segmentation. For 14 fetal MR scans with a gestational age distribution between 20.57 and 22.86 weeks, a probabilistic map of the tissue distribution was constructed to segment white matter, gray matter, germinal matrix, and right cerebrospinal fluid using an expectation maximization (EM) model approach. Before segmentation, a bias field correction was performed using another EM model. Later, considering the extension of this framework to a wider range of gestational ages, the authors proposed another temporal model to construct a spatiotemporal atlas of the fetal brain [8], which was created using polynomial simulations of the changes in MR intensity, tissue probability and shape from a group of fetal MRIs between 20.57 and 24.71 weeks of gestational age. Serag et al. [9] proposed a method to construct a 4D multichannel atlas of the fetal brain that utilized the nonrigid registration of MR brain images and included the average intensity template of multiple modalities and tissue probability maps. Compared with the probability maps generated by the affine registration method, the tissue probability map generated by this method improved the automatic segmentation process based on the atlas. The data used in the study were collected from MR images of 80 fetuses aged between 23 and 37 weeks of gestational age (GA). Wright et al. [10] used MR images of 80 developing fetuses in the gestational age range of 21.7 to 38.9 weeks, took the spatiotemporal atlas as tissue priors, and adopted the Markov random field regularized expectation maximization (EM-MRF) method proposed by Ledig et al. [11] to improve tissue segmentation accuracy. The technique of atlas fusion was used, while the SVR technique was employed before extracting the brain to alleviate the sensitivity of motion-impaired fetal images to alignment.

In recent years, deep learning methods, particularly convolutional neural networks (CNNs), have gained popularity for many target recognition and biological image segmentation challenges [12,13,14]. Unlike traditional classification methods, CNN automatically learns complex and representative features directly from the data, eliminating the need to first extract a set of handcrafted features from the image as input to a classifier or model [15]. Due to this property, research on CNN-based fetal brain tissue segmentation has mainly focused on the design of network architectures rather than on extracting features in image processing. As a result, deep learning methods tend to achieve better performance than traditional machine learning methods. Kelly et al. first used three 2D U-Nets trained in three directions (axial, sagittal, and coronal) to form three separate networks and then trained a 3D U-Net with the same structure as the 2D U-Net. Data augmentation, epochs, loss functions, and optimizers were the same. Finally, they evaluated each of these four networks. Khalili et al. [16] first used a CNN to extract intracranial volumes by automatically cropping the image to the region of interest, after which a CNN with the same structure was used to segment the extracted volumes into seven brain tissue categories. Asim lqbal et al. [17] transformed 3D brain stacks into 2D brain images (256 × 256) covering the top-down axial plane to make the dataset compatible with SeBRe. Transfer learning was used during their training process. The FeTA brain dataset is initialized with pretrained weights for the Microsoft COCO dataset.

Different congenital diseases lead to various changes in fetal brain tissues, so this research aimed to help doctors obtain more intelligent and rapid structural segmentation to provide quantitative information for auxiliary diagnosis of congenital diseases. The fetal brain is small, with irregular and interconnected tissues. Automatic segmentation algorithms can reduce the burden of manual segmentation, improve segmentation efficiency, and quickly identify various tissues, which is beneficial to the judgment of fetal congenital diseases. In medical image processing, automatic segmentation algorithms usually use the U-Net model, which mainly differs in image pre-processing and post- processing, as well as the fine-tuning and parameter setting during the training process. However, the traditional U-Net architecture has several drawbacks, such as (1) the feature extraction capability being insufficient and (2) the inability to use global contextual information. Therefore, focusing on these two problems, the main contribution of this work is to design a new network structure, which combines CNN and transformer. The convolution layer in CNN obtains the output features through the convolution core, but the receptive field of the convolution core is small. It is not efficient to simply increase the receptive field by stacking convolution layers. The self-attention mechanism in transformer facilitates capturing the global information to obtain a larger receptive field. Therefore, we combined CNN and self-attention to design a feature pyramid model based on the contextual transformer block (CoT-Block). It integrated context information mining and self-attention learning into a unified architecture to guide the learning of dynamic attention matrix, thus enhancing the ability of image feature extraction. At the same time, the introduction of the hybrid dilated convolution module can expand the receptive field and retain detailed spatial information, effectively extracting the global context information of fetal brain MRI tissues. In this way, the defects of the traditional U-Net model structure mentioned above can be compensated to a certain extent. Comparative experiments with several state-of-the-art models showed that our trained model was less computationally intensive, faster, and had the best segmentation performance overall.

## 2. Materials and Methods

### 2.1. Fetal MRI Data

This study included T2-weighted MRI scans of 80 fetuses at a gestational age between 20 and 35 weeks. These data [18] were acquired on 1.5 T and 3 T GE clinical whole-body scanners (Signa Discovery MR450 and MR750) using either an 8-channel cardiac coil or a body coil. Several T2-weighted single-shot fast spin echo (ssFSE) images with a resolution of 0.5 mm × 0.5 mm × 3 mm were acquired in all three planes (at least one image each in the axial, sagittal, and coronal planes) for each subject and reconstructed as a single high-resolution volume of 0.5 mm × 0.5 mm × 0.5 mm using the MIAL Superresolution Toolkit [19,20] and Simple IRTK [21]. The repetition time (TR) was set to 2000–3500 ms, the echo time (TE) was set to 120 ms (minimum), the flip angle was set to 90 degrees, and the sampling percentage was 55%. Field of view (200–240 mm) and image matrix (1.5 T: 256 × 224; 3 T: 320 × 224) were adjusted depending on the gestational age and fetal size. The imaging plane was oriented relative to the fetal brain, and axial, coronal and sagittal images were acquired.

**Figure 1:**
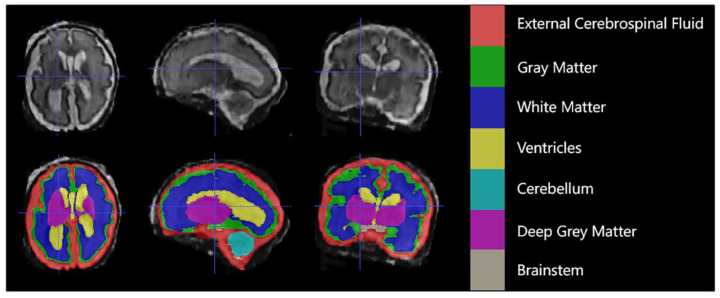
An example of manual segmentation (red: external cerebrospinal fluid; green: GM; dark blue: WM; yellow: ventricles; cyan: cerebellum; maroon: deep GM: gray: brainstem).

Each case consisted of a 3D superresolution reconstruction of the fetal brain (256 × 256 × 256 voxels). Training cases had an annotated label map corresponding to 7 different brain tissue types: external cerebrospinal fluid (eCSF), gray matter (GM), white matter (WM), lateral ventricles (LV), cerebellum (CB), deep gray matter (DGM), and brainstem (BS). As shown in Figure 1 below.

### 2.2. Model Architecture

**Figure 2:**
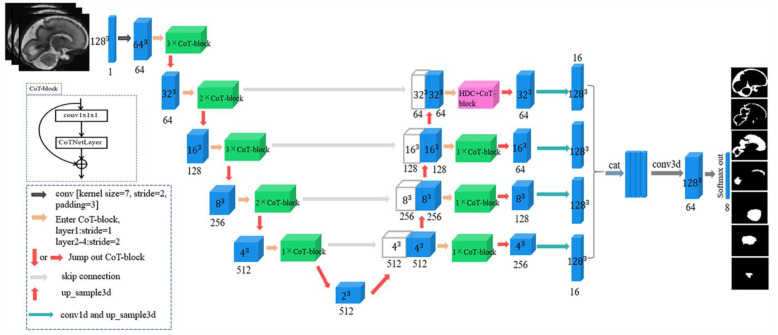
Network architecture: the network consists of a backbone network, which is composed of an encoder–decoder architecture, skip connection, and feature combination.

We combined CNN with Transformer to design a network model as shown in Figure 2. The backbone network of this model consisted of four parts: encoder, decoder, skip connection, and feature combination. Each layer of the encoder introduced self-attention blocks with the number of 3-2-3-2-1, and each layer of the decoder introduced one self-attention block and added a hybrid dilated convolution in the last layer. For localization, each feature map in the decoder was connected with the corresponding feature map from the encoder, and this structure was the skip connection. It linked the low-level features to the high-level features and collected the detailed information lost in the encoder, thus obtaining more accurate spatial information and improving the quality of the final tissues contour prediction.

#### 2.2.1. Backbone Network

Part of the encoder followed the new architecture of the contextual transformer block (CoT-Block), which will be explained in detail in the subsequent sections. The network started by extracting a series of feature maps at different resolutions. The shallower feature maps contained the high-resolution detailed information needed to correctly depict the tissue boundaries, and the deeper feature maps contained both coarse- and high-level information to help predict the overall profile of the tissue. The input was first processed by a 7 × 7 × 7 convolution layer with a stride of 2 and padding of 3. Then, batch normalization (BN) and a rectified linear unit (ReLU) were used, followed by a 3 × 3 × 3 max pooling operation with stride 2 for downsampling. The number of feature maps in the encoder part of the network was 64, 128, 256, 512, and 512, where the number of feature channels was doubled in each downsampling step.

Similarly, the decoder was also composed of a CoT-block. Each step consisted of up-sampling the feature maps and converting them to the size of the input image using trilinear interpolation, then halving the number of feature channels and concatenating them with the corresponding feature maps in the encoder. This large number of feature channels in the up-sampling section allowed the network to propagate contextual information to the higher resolution layers. Then, a convolution operation with a kernel size of 3 × 3 × 3 was applied to each layer of the output feature map to change its channel, and trilinear interpolation was applied to alter its size, creating 16 features in each feature map. Finally, the resultant feature maps were merged via concatenation, and these feature maps went through 3 × 3 × 3 convolutional layers followed by BN and PReLU to generate the probability maps of seven brain tissues.

#### 2.2.2. Hybrid Dilated Convolution

In the last layer of the decoder, a hybrid dilated convolution module [22] was introduced, which consisted of three 3D dilated convolution branches with dilation rates of d = 1, 2, and 4. This module expanded the receptive field of the convolution kernel while keeping the number of parameters constant so that each convolution output contained a large range of information; at the same time, it ensured that the size of the output feature map remained unchanged.

#### 2.2.3. CoT-Block

**Figure 3:**
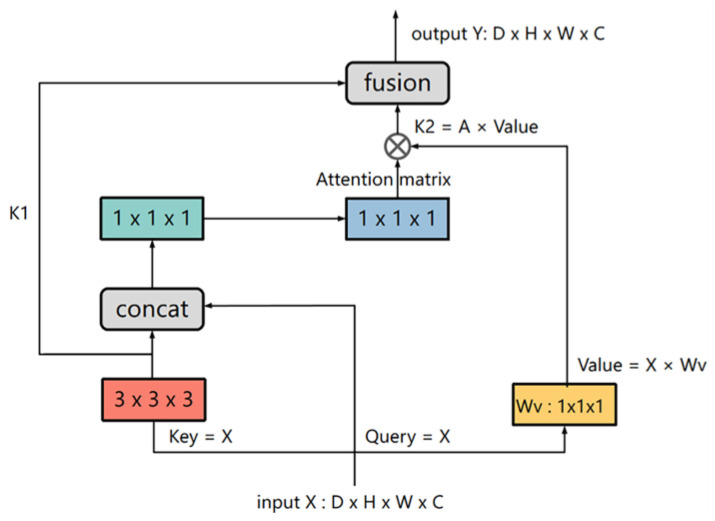
Contextual Transformer block (CoT-Block), a novel attention structure [23].

The contextual transformer block (CoT-Block) is a novel attention structure that makes full use of the context information of the key to guide the learning of the dynamic attention matrix to enhance the ability of image feature representation. Its structure is shown in Figure 3.

The conventional self-attention block transformed the input feature map *X* into queries *Q = XW_q_*, keys *K = XW_k_*, and values *V = XW_v_* via the embedding matrix (*W_q_,W_k_,W_v_*), respectively. When the image was 3D, each embedding matrix (*W_q_, W_k_, W_v_*) adopted a 1 × 1 × 1 convolution in space. Then, keys *K* and queries *Q* performed matrix multiplication to obtain the local spatial relationship *R*, which was expressed as follows: R=K×Q, and further enriched the local relation matrix *R* through position information *P*: $\hat{R}=R+P\times Q$. However, this traditional self-attention mechanism has a defect, that is, when we study a pixel, we get keys *K* from the surrounding pixels to do correlation calculation with this pixel’s query *Q*, so all *Q-K* relationships are learned independently, without making use of the rich context information between pixels. This will severely limit the capacity of self-attention learning of feature map in the training process. For this reason, Li et al. [23] proposed the contextual transformer block (CoT-Block), a novel module that integrated contextual information mining and self-attention learning into a unified architecture. First of all, instead of directly converting the input feature map through the embedded matrix, it first used 3 × 3 × 3 convolution to encode the keys *K* of surrounding pixels to obtain the static context information $K_{1}$ of local adjacent positions. Then, it concatenated $K_{1}$ and query *Q* of the pixel being studied into two consecutive 1 × 1 × 1 convolutions to learn the dynamic multi-head attention matrix, which we can represent as $W_{\theta }$ and $W_{\delta }$. Attention matrix *A* was expressed as $A=\left[K_{1},\;Q\right]\;W_{\theta }W_{\delta }$. Instead of obtaining isolated *Q-K* pairs, this process obtained rich contextual information representation between pixels and learnt more about the details of the tissue. Then, the attention matrix *A* was multiplied by values *V* to obtain$\;K_{2}$: $K_{2}=A\times V$, which implemented dynamic context representation of input. Finally, the module fused static and dynamic context representation to get the output result.

### 2.3. Loss Function

We used the combination of Dice loss and cross-entropy loss to train our network. The Dice coefficient is a similarity set metric function named by Lee Raymond Dice, which is commonly used to calculate the similarity between two samples. Its value ranged from 0 to 1. The Dice coefficient D between two binary volumes was denoted by

(1) $$D=\frac{2\sum_{i}^{N}p_{i}g_{i}}{\sum_{i}^{N}p_{i}^{2}+\sum_{i}^{N}g_{i}^{2}}$$

where the sums of the predicted binary segmentation volume $p_{i}\;\in P$ and the ground truth binary volume $g_{i}\;\in G$ were spread across the N voxels. The Dice loss was expressed as $L_{dice}=1-D$.

In dichotomous models, such as logistic regression and neural networks, the ground truth was labeled [0, 1], representing negative and positive classes, respectively. The model usually ended up with a sigmoid function (softmax function in the case of multiple classifications), which outputs a probability value reflecting the likelihood of predicting a positive class. The sigmoid function was expressed as follows: $\sigma \left(x\right)=\frac{1}{1+e^{-x}}$. The cross-entropy loss function was:

(2) $$L=\frac{1}{N}\sum_{i}L_{i}=\frac{1}{N}\;\sum_{i=1}^{N}-[y_{i}\;log\;p_{i}+\left(1-y_{i}\right)\log\left(1-p_{i}\right)]$$

In this equation, $y_{i}$ and $p_{i}$ denote the ground truth brain tissue map and the predicted brain tissue probability map.

### 2.4. Implementation and Training

We implemented our network in PyTorch [24] and trained it in an end-to-end manner using the Adam optimizer [25] with a weight decay of 1 × 10^−4^. We used an initial learning rate of 1 × 10^−4^ and reduced it by a factor of 0.7 after 30 training epochs. Learning stopped after 300 epochs. All the training and experiments were run on a standard workstation equipped with 32 GB of memory, an Intel (R) Core (TM) i7-10700 CPU working at 2.90 GHz, and an Nvidia GeForce RTX 3090 GPU with a batch size of 2.

### 2.5. Data Augmentation

We applied a strong random data augmentation method to our training data, specifically applying random rotation of −15° to 15°, random horizontal flipping, translation transformation (10 voxels), and random 3D elastic transformation. We have conducted 300 epochs of training. At each training epoch, a different random augmentation transformation was applied than previous ones, so we ended up with 300 different representations of the data.

## 3. Experiments and Results

### 3.1. Alternative Techniques

To verify the segmentation performance of our proposed method, we compared the model with three existing deep neural networks, namely, 3D U-Net [26], V-Net [27], and 3D DMFNet [28]. They were both 3D network structures used to process medical images.

3D U-Net extends the U-Net architecture proposed by Ronneberger et al. [29]. The highlight of the current architecture is that it can be trained from scratch on sparsely annotated volumes and works on arbitrarily large volumes due to its seamless tiling strategy. It has been shown to be a powerful segmentation CNN with success in multiple segmentation tasks [30,31,32].

V-Net is a variant of U-Net. The left part consists of a compression path, while the right part decompresses the signal until its original size is reached. Convolutions are all applied with appropriate padding. The greatest difference with U-Net is that in each stage, it learns a residual function, which ensures convergence in a fraction of the time required by a similar network that does not learn residual functions, thus becoming the greatest improvement of V-Net.

The 3D dilated multifiber network (DMFNet) builds upon the multifiber unit [33], which uses efficient group convolution and introduces a weighted 3D dilated convolution operation to gain multiscale image representation for segmentation.

### 3.2. Gestational Age Analysis

We analyzed the effect of different gestational ages on algorithm performance. The fetal brain undergoes a variety of changes throughout development, such as increased brain volume, neuronal migration, and synaptogenesis. As a result, tissue contrast changes dramatically, particularly in the cortex. For example, as gestational age increases, the brain gyrus in the group 28–35 GW becomes narrower and the border between white matter and gray matter becomes blurred. Partial volume effects caused by tall and narrow gyri result in poor gray matter/white matter border visibility in certain regions [34]. Therefore, the random error in segmentation of gray matter and white matter between 28–35 GW may increase. The lateral ventricles change from the fetal type (vesicular and bicornuate) to the adult type with increasing gestational age, and the volume ratio of the lateral ventricles to brain increases between 10 and 13 GW, corresponding to the blistering phase of the ventricular system. At 28–35 weeks, the thickness of the hemispheric mantle increases exponentially, resulting in a decrease in the volume ratio of the lateral ventricles to brain [35]. For our study, we used 80 fetal MR scans with gestational ages ranging from 20 to 35 weeks. We divided these images into two age groups (20–27 weeks and 28–35 weeks) and analyzed the impact of different gestational ages on the performance of the segmentation algorithm through comparison experiments between these two groups.

### 3.3. Evaluation Metrics

The dataset was divided into training set, validation set and test set according to the ratio of 6:1:1. In the training and validation sets, we adopted ten-fold cross-validation to ensure the reliability of the results. We used six evaluation metrics to evaluate performance of the models: (1) the Dice similarity coefficient (DSC) was used to measure the similarity between Ground Truth and Prediction, (2) the segmentation precision (PRE), (3) the segmentation sensitivity (SEN), (4) the segmentation specificity (SPC), (5) the Volume Similarity (VS) was used to compared the volumes of the two segmentations, and (6) Hausdorff95 Distance (HD95) was defined as the quantized value of 95% of the maximum distance of the surface distance between Ground Truth and Prediction. All the evaluation metrics were calculated volume by volume, and each volume represented a specific tissue region of each patient.

(3) $$DSC=\frac{2TP}{2TP+FN+FP}$$

(4) $$PRE=\frac{TP}{TP+FP}\;$$

(5) $$SEN=\frac{TP}{TP+FN}$$

(6) $$SPC=\frac{TN}{TN+FP}$$

(7) $$\mathrm{VS}=1-\frac{\left|X-Y\right|}{X+Y}$$

(8) $$\mathrm{HD}95=max_{k95\%}\left[d\left(X,Y\right),d\left(Y,X\right)\right]\;$$

In the above equations, *TP*, *FP*, *TN*, *FN* represent the value of true positives, false-positives, true negatives, and false negatives, respectively. *X* and *Y* represent the Prediction and Group Truth, respectively. The lower the HD95 value, the higher the segmentation accuracy. The larger the other values, the better the segmentation performance. In addition, we also test the model’s parameters and floating point operations (FLOPs). The smaller the parameters and FLOPs, the less computational resources the model requires.

### 3.4. Evaluation Results

**Figure 4:**
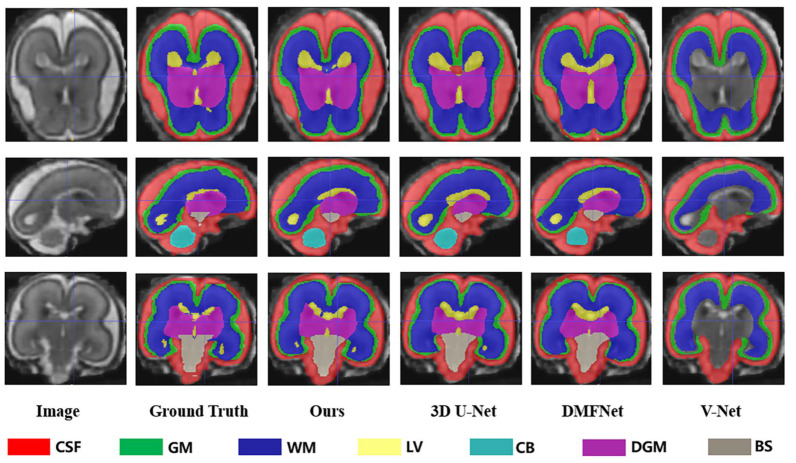
Segmentation results of different methods (Ours, 3DU-Net, DMFNet and V-Net) in axial, sagittal and coronal planes respectively.

**Figure 5:**
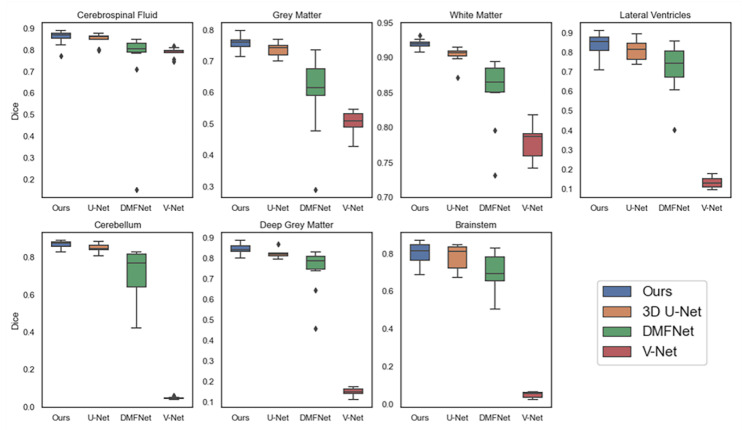
Analysis of dice values of four different methods (Ours, 3DU-Net, DMFNet and V-Net) on seven tissues. The rhomb indicates the abnormal value.

As demonstrated in Table 1, based on all evaluation metrics, overall, our method outperformed the other methods with the best average segmentation on 7 tissues. DSC, VS, PRE, SEN, and SPC all achieved the highest values with (83.79 ± 3.36)%, (84.84 ± 3.23)%, (85.73 ± 3.75)%, (84.32 ± 5.22)%, and (99.33 ± 0.15)%, and HD95 achieved the smallest with (35.66 ± 2.07) mm. In terms of different tissues, our method got better evaluation metrics in cerebrospinal fluid, gray matter, ventricle, cerebellum, and brainstem, whereas the best segmentation on white matter and dark gray matter were obtained with 3D U-Net. Unexpectedly, the ventricles, cerebellum, deep gray matter, and brainstem were not fully segmented in V-Net. We showed examples for visual comparison in Figure 4. Taking the ventricle as an example, it can be seen that only our method better identified the boundary between it and other tissues, such as white matter and deep gray matter, which was the result closest to the ground truth, whereas other methods either blurred the boundary or failed to find the ventricle part. Figure 5 depicted box plots to further visualize the differences in the Dice results. We have seen that the segmentation effect of the four models on CSF was relatively good, and Dice values were concentrated in a high range (0.8–0.9). For gray matter and white matter segmentation, the interquartile range (IQR) of DMFNet and V-Net was large, indicating a more dispersed distribution of Dice values. Table 2 showed the number of parameters and FLOPs for the four models. Our parameters were 24.21 M smaller than 3D U-Net, and FLOPs were reduced by 85.94%. DMFNET had the lowest number of parameters and FLOPs, but its segmentation performance was much lower than our method and 3D U-Net. Therefore, combining the results in Table 1 and Table 2 demonstrated that our network was a better model in the comprehensive evaluation of segmentation performance and computing resource utilization.

**Figure 6:**
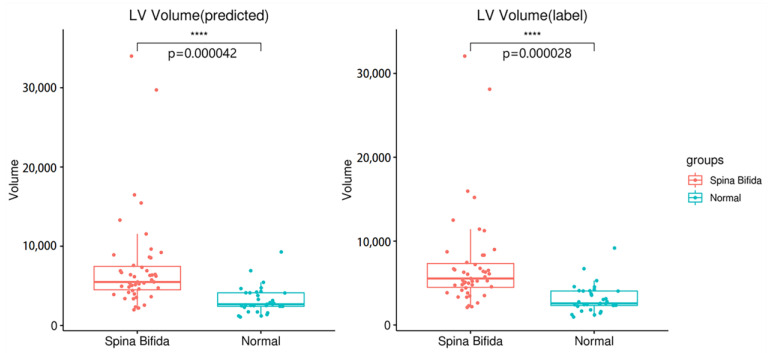
Analysis of ventricular volumes in the normal group and pathological group (spina bifida). **** represents *p* value less than 0.0001.

In the Introduction section above, we learned that congenital disorder may lead to enlarged ventricles. Here, we divided the data into the normal group and the pathological group (with spina bifida), then segmented the two groups with our network structure and calculated the volume of the ventricles. The volumes of the two groups were shown in Figure 6. The comparison between groups showed that the ventricle in the pathological group was significantly enlarged compared with the normal group (*p* < 0.0001), suggesting that measuring the changes in ventricular volume through fetal brain MRI tissue segmentation can be used as an important reference for diagnosing related congenital diseases.

**Figure 7:**
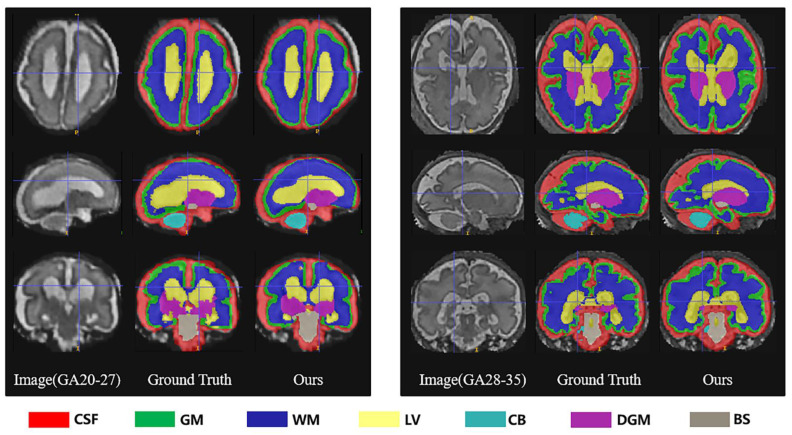
Segmentation results of different gestational age groups (20–27 weeks and 28–35 weeks) in axial, sagittal and coronal planes respectively.

Table 3 showed the evaluation results of several metrics for two different gestational age groups (20–27 GW and 28–35 GW) as a way to analyze the effect of gestational age on the performance of our segmentation algorithm. The results obtained in the table showed that in the 28–35-week group, the segmentation performance of both gray matter and white matter became worse, with a significant decrease in DSC, VS, PRE, and SEN and an increase in HD95. The segmentation of the lateral ventricles was also poorer in the 28–35-week group. This supported our hypothesis that segmentation was more difficult due to the decrease in ventricle-to-brain volume ratio with increasing gestational age. Overall, the 28–35 age group showed a slight increase in segmentation difficulty compared to the group of 20–27 GW, with a 2.15% decrease in mean DSC (%) from 89.88 to 87.95 and a 1.98% decrease in VS (%) from 91.58 to 89.77. We also provided a few examples for visual comparison of the two age groups in Figure 7. The cerebral cortex expanded with increasing gestational age, and the morphology of the cerebral gyri and sulci became more notable. Discernibility of the gray/white matter boundary became variable in some regions due to partial volume effects produced by gyri. Gray matter (green) segmentation became more indistinguishable from cerebrospinal fluid (red) and white matter (dark blue) boundaries. In the 28–35 GW group, the volume of ventricles (yellow) became significantly smaller and the shape changed, and it was no longer simply vesicular or bicornuate, which also led to an increase in the random error of segmentation.

## 4. Discussion

Congenital diseases occur during the fetal period, which is caused by abnormal development of the fetus due to external or internal adverse factors during its growth and development in the womb. Once congenital diseases appear, they will bring serious burden to families and society. Timely detection and treatment of fetal congenital diseases will help to reduce mortality. Therefore, it is essential to conduct regular health analysies of developing fetus before delivery. Doctors usually analyze health conditions through changes in fetal brain tissues. Fetal MRI scanning has been an important auxiliary tool.

However, the fetal brain is small and has a complex tissue structure and the intensity of different tissues is not uniform but varies gradually across the image space. Higher field strength scanners result in greater intensity variation. The partial volume (PV) effect of mixing different tissues in a single voxel poses additional difficulties for accurately depicting tissue boundaries, such as the mixing of CSF and GM on the CSF-CGM (cortical GM) boundary results in an intensity similar to that of WM. This PV effect causes the CSF-CGM to mislabel voxels as WM. In addition to lower contrast, tissues such as the cerebellum and brainstem may have issues with network architecture not fully extracting features due to their smaller distribution. Therefore, to help physicians analyze tissue changes more quickly and accurately to determine congenital diseases, it is necessary to design a network structure with stronger feature extraction capability that can analyze global contextual information of tissues and perform accurate segmentation in a short period of time.

V-Net is a network modified from U-Net, which was first proposed specifically for medical image analysis. Although it demonstrated good segmentation performance on prostate MR images, it did not perform well in our fetal MRI tissue segmentation, where it completely failed in segmentation tasks of the cerebellum, deep gray matter, and brainstem. The possible reason was that it did not make full use of the global context information of various tissue structures, so it was not strong enough in feature extraction. DMFNet, relative to V-Net, was able to segment the above three brain tissues better because the 3D-dilated convolution introduced in DMFNet can build multiscale feature representations and help extract smaller tissue patches, and this module was also used in our network. In previous studies on fetal MRI brain tissue segmentation, the most popular convolutional neural network U-Net was commonly used to form a 2D network in three directions or directly using a 3D network. The 3D U-Net is a powerful model. It can segment all seven tissues better than V-Net and DMFNet in our tasks. However, it has too many parameters and takes too long to run for a quick diagnosis. Moreover, a major feature of medical images is the small amount of data. Therefore, if the network structure is too complex and there are too many parameters, the trained model may suffer from overfitting and bias.

Therefore, for the three requirements of (1) better feature extraction capability, (2) using global contextual information, and (3) fewer parameters and shorter training time, we designed a lightweight tissue segmentation model that combined CNN and the self-attention mechanism by introducing the contextual transformer block (CoT-Block), a novel attentional structure block, at each layer of the encoder and decoder. Previous studies have proposed different attention mechanisms for applications such as image classification and image segmentation. These attention mechanisms came in different forms, such as spatial attention, channel attention, and self-attention. The module we introduced in the proposed model was the self-attention module. The original self-attention mechanism in the NLP field mainly solves the long-distance dependence problem by computing the interactions between words, and extending to the field of computer vision, the research objects are not sequences but large pixel matrices, but they have the common feature that the input samples contain information of multiple elements at the same time, and these information are not independent, but have different degrees of contact, and are mutually contextual information. In vision domain, a simple migration of the self-attention mechanism from NLP to CV is to directly perform self-attention over feature vectors across different spatial locations within an image, and let each element in the input vector extract useful information from each other in turn before inputting it to the subsequent network for computation. Most existing techniques directly utilize the traditional self-attentive mechanism, ignoring the explicit modeling of rich contexts among neighbor keys, which severely limits the ability of self-attention learning of feature maps in the training process. In contrast, we first used the 3 × 3 × 3 convolution to encode the context of surrounding voxels to obtain static context information of local adjacent positions to guide the learning of dynamic attention matrix. We used this structure block to replace a 3 × 3 × 3 convolution in each layer of the encoder and decoder, which improved the representational properties of the deep network and enhanced the ability of the model to extract features from different fetal tissues. In addition, in the last layer of the decoder, a hybrid dilated convolution module was introduced, based on which the receptive field can be expanded to obtain global contextual information in fetal MRI and capture the multi-scale 3D spatial correlation of tissues, which facilitated the segmentation of smaller tissues such as cerebellum and brainstem and improved the accuracy of segmentation. The overall parameter size of our model was 66.09 M, and the time per training epoch was 33 s, which was a superior algorithm with less space complexity and time complexity compared to 90.3 M and 90 s of 3D U-Net.

We also analyzed the effect of different gestational ages on algorithm performance. The fetal brain experienced various changes throughout development. As gestational age increases, the cerebral cortex expands, the morphology of the gyri and sulci becomes more obvious, and the narrowed gyrus makes it more difficult to distinguish between white matter and gray matter boundaries, increasing random errors. The increase of gestational age also causes the ventricles to change from fetal (vesicular and bicornuate) to adult type, becoming smaller in size, while the thickness of the hemispheric mantle increases exponentially, so their volume ratio decreases and segmentation becomes more difficult. For the hypothesis that congenital diseases cause tissue changes, we conducted a verification experiment to analyze the tissues characteristics of normal brain and pathological brain. We divided the data into a normal group and a pathological group with spina bifida. We calculated the ventricle volumes based on the segmentation results of the two groups. The comparison showed that there was a significant difference in the volume of the ventricles between the pathological group and the normal group. This showed that fetal brain MRI tissue changes can be used as a way to diagnose congenital diseases.

We used 3D convolutional kernels to build our 3D network. To reduce the number of computations as much as possible, we cropped the image; only the excess background was cropped to preserve the intact fetal brain part, and it was scaled to 128 × 128 × 128. Additionally, we tried different choices of batchsize = 2, 4, 5, and 8, and the experimental results showed that the best segmentation result was obtained with batchsize = 2.

## 5. Conclusions

In this work, we proposed an automatic segmentation algorithm for fetal brain MRI, which aimed to segment seven tissues of the fetal brain to help diagnose congenital diseases or evaluate the effect of an intervention or treatment. We introduced a novel network structure, a feature pyramid model based on the contextual transformer block, which introduced the CoT-Block in both the encoder and decoder, which integrated contextual information mining and self-attention learning into a unified architecture to guide the learning of the dynamic attention matrix, thus enhancing the image feature representation capabilities. In addition, a hybrid dilated convolution module was also introduced in the last layer of the decoder, which can expand the receptive field and retain detailed spatial information to effectively extract global contextual information in medical images. The comparison experiments with other network models revealed that our model outperformed several advanced deep learning network models.

Due to the small amount of data in this work, in the subsequent studies, we will collect as much fetal brain data as possible from different institutions for a more detailed analysis, or introduce transfer learning to increase training samples to further verify the performance of our algorithm. In addition, during the data acquisition process, the fetal position in the maternal body is constantly changing, with no regularity, and motion artifacts are inevitable. Therefore, in order to address the artifact problems such as intensity nonuniformity generated during the scanning process, an adversarial data augmentation technique can be used to train the neural network by simulating the intensity inhomogeneity (bias field) caused by the common artifacts in MRI, which can improve the generalization ability and robustness of the model and can alleviate the problem of data scarcity. We can segment the brain into seven different tissue structures, allowing a detailed brain tissue analysis for a specific congenital disease, better predicting outcomes of surgery, such as fetal patients with spina bifida, and we can reconstruct the fetal brain at pre- and post- treatment time points and train our model to automatically segment tissues, register the post-treatment image to the pre-treatment image to determine tissue changes between the two time points, analyze the lesion size, type, and the effects of gestational age at the time of treatment on various tissue morphological changes, to better predict the surgical outcomes to provide guidance for prenatal parents.

## Figures and Tables

**Table 1:** Comparison of segmentation results of different methods (V-Net, DMFNet, 3DU-Net and Ours) on different tissues based on quantitative performance metrics (DSC, VS, HD95, PRE, SEN, SPC). The best value for each metric in each tissue is marked in bold.

Method	Metrics	eCSF	GM	WM	LV	CB	dGM	BS	Mean ± SD
V-Net	DSC (%)	64.32 ± 2.45	32.36 ± 4.63	80.89 ± 2.30	8.87 ± 3.35	5.44 ± 1.75	9.76 ± 3.12	4.57 ± 1.49	29.46 ± 2.73
	VS (%)	72.66 ± 2.59	21.75 ± 11.83	88.32 ± 3.09	-	-	-	-	-
	HD95 (mm)	22.69 ± 3.17	25.86 ± 2.44	26.81 ± 2.35	37.17 ± 1.53	**53.48** ± **2.61**	43.02 ± 4.53	**48.26** ± **3.50**	36.76 ± 2.88
	PRE (%)	69.64 ± 2.37	**88.29** ± **8.20**	83.81 ± 4.40	-	-	-	-	-
	SEN (%)	77.45 ± 1.52	13.09 ± 7.85	93.49 ± 3.24	-	-	-	-	-
	SPC (%)	97.73 ± 0.63	**99.90** ± **0.09**	97.74 ± 0.71	-	-	-	-	-
DMFNet	DSC (%)	73.59 ± 2.3	59.73 ± 7.05	85.21 ± 3.47	74.28 ± 5.40	73.05 ± 4.11	76.05 ± 2.55	69.79 ± 4.89	73.10 ± 4.25
	VS (%)	74.10 ± 2.12	60.87 ± 6.94	85.68 ± 3.48	75.67 ± 4.74	74.05 ± 3.85	76.79 ± 2.63	71.10 ± 4.60	74.04 ± 3.77
	HD95 (mm)	21.33 ± 1.69	23.71 ± 1.29	**25.33** ± **1.17**	35.35 ± 1.93	54.79 ± 2.92	41.03 ± 1.34	49.26 ± 3.69	35.83 ± 2.00
	PRE (%)	73.76 ± 3.62	62.04 ± 9.47	84.41 ± 4.79	74.84 ± 7.3	82.31 ± 6.99	80.47 ± 8.64	70.66 ± 8.1	75.50 ± 6.99
	SEN (%)	75.67 ± 2.90	60.22 ± 6.18	87.19 ± 3.86	79.39 ± 5.95	70.79 ± 8.08	78.13 ± 9.28	75.77 ± 5.31	75.31 ± 5.94
	SPC (%)	97.41 ± 0.39	98.12 ± 0.44	97.96 ± 0.83	99.56 ± 0.09	99.87 ± 0.05	99.73 ± 0.12	99.80 ± 0.07	98.92 ± 0.28
3D U-Net	DSC (%)	80.19 ± 2.83	69.04 ± 4.96	**89.94** ± **2.48**	85.54 ± 4.21	82.50 ± 4.47	**85.04** ± **3.35**	78.08 ± 4.89	81.48 ± 3.88
	VS (%)	80.43 ± 2.84	69.41 ± 4.01	**90.15** ± **2.47**	86.27 ± 6.86	83.97 ± 4.54	**85.78** ± **3.36**	80.10 ± 4.46	82.30 ± 4.08
	HD95 (mm)	22.56 ± 3.20	24.80 ± 1.44	26.48 ± 1.40	35.48 ± 2.13	56.80 ± 4.28	**40.12** ± **2.40**	51.77 ± 3.80	36.06 ± 2.66
	PRE (%)	82.06 ± 2.90	67.87 ± 3.03	89.36 ± 4.46	83.77 ± 8.41	82.02 ± 1.56	**91.28** ± **3.75**	78.09 ± 8.94	82.06 ± 4.72
	SEN (%)	79.18 ± 2.36	71.48 ± 6.90	**91.10** ± **2.02**	**89.04** ± **5.13**	**89.29** ± **10.6**	81.40 ± 7.06	83.58 ± 6.64	83.58 ± 5.82
	SPC (%)	**98.57** ± **0.78**	98.54 ± 0.43	98.80 ± 0.64	99.71 ± 0.09	99.87 ± 0.14	**99.87** ± **0.07**	**99.90** ± **0.03**	99.32 ± 0.31
Ours	DSC (%)	**85.49** ± **3.16**	**71.20** ± **4.87**	89.73 ± 1.73	**86.38** ± **4.78**	**85.75** ± **4.42**	84.64 ± 2.91	**83.31** ± **1.62**	**83.79** ± **3.36**
	VS (%)	**85.88** ± **3.13**	**72.01** ± **4.84**	90.06 ± 1.70	**87.22** ± **4.52**	**87.65** ± **4.17**	85.53 ± 2.97	**85.56** ± **1.26**	**84.84** ± **3.23**
	HD95 (mm)	**20.79** ± **1.77**	**23.67** ± **1.52**	25.38 ± 1.41	**34.00** ± **1.84**	54.91 ± 2.92	41.51 ± 1.34	49.34 ± 3.69	**35.66** ± **2.07**
	PRE (%)	**85.58** ± **4.03**	72.34 ± 3.39	**90.41** ± **2.95**	**88.76** ± **4.25**	**91.83** ± **0.98**	84.65 ± 4.88	**86.52** ± **5.75**	**85.73** ± **3.75**
	SEN (%)	**86.23** ± **2.70**	**71.84** ± **6.74**	89.84 ± 2.63	85.90 ± 6.23	84.20 ± 7.53	**87.04** ± **6.34**	**85.19** ± **4.34**	**84.32** ± **5.22**
	SPC (%)	98.26 ± 0.36	98.64 ± 0.16	**98.95** ± **0.28**	**99.75** ± **0.14**	**99.95** ± **0.02**	99.82 ± 0.05	99.93 ± 0.02	**99.33** ± **0.15**

**Table 2:** Shows the comparison of Param, FLOPs of different methods (V-Net, DMFNet, 3DU-Net and Ours).

Method	Param (M)	FLOPs (G)
V-Net	45.63	809.78
DMFNet	3.87	26.92
3D U-Net	90.3	2128.3
Ours	66.09	299.24

**Table 3:** Comparison of segmentation results of different gestational age groups (20–27 weeks and 28–35 weeks) based on quantitative performance metrics (DSC, VS, HD95, PRE, SEN, SPC).

GA	Metrics	eCSF	GM	WM	LV	CB	dGM	BS	Mean±SD
20–27	DSC (%)	87.49 ± 8.31	83.61 ± 1.29	95.66 ± 0.43	90.59 ± 3.29	92.13 ± 0.29	94.37 ± 0.50	85.30 ± 2.79	89.88 ± 2.41
	VS (%)	89.05 ± 6.94	85.46 ± 1.24	96.18 ± 0.40	91.82 ± 2.72	94.08 ± 0.31	95.44 ± 0.49	89.00 ± 1.78	91.58 ± 1.98
	HD95 (mm)	22.64 ± 3.08	22.91 ± 1.38	24.39 ± 1.52	33.52 ± 2.03	55.97 ± 2.55	40.82 ± 1.58	52.54 ± 3.18	36.11 ± 2.19
	PRE (%)	91.48 ± 3.32	85.78 ± 0.81	96.26 ± 0.30	90.72 ± 2.62	94.58 ± 0.71	96.23 ± 0.92	89.36 ± 0.74	92.06 ± 1.35
	SEN (%)	87.01 ± 0.10	85.14 ± 1.82	96.11 ± 0.51	92.94 ± 2.88	93.59 ± 0.84	94.68 ± 0.27	88.67 ± 2.79	91.16 ± 1.32
	SPC (%)	99.44 ± 0.35	99.43 ± 0.05	99.45 ± 0.19	99.77 ± 0.14	99.96 ± 0.01	99.94 ± 0.01	99.96 ± 0.01	99.71 ± 0.11
28–35	DSC (%)	92.04 ± 0.88	76.41 ± 3.86	94.68 ± 0.70	77.98 ± 3.93	93.09 ± 0.68	94.27 ± 0.61	87.17 ± 1.68	87.95 ± 1.76
	VS (%)	92.78 ± 0.87	78.38 ± 3.56	95.23 ± 0.65	81.35 ± 3.55	94.70 ± 0.70	95.38 ± 0.59	90.59 ± 1.56	89.77 ± 1.64
	HD95 (mm)	20.50 ± 1.83	23.47 ± 1.36	24.69 ± 1.35	37.26 ± 2.43	53.71 ± 1.96	39.77 ± 1.65	50.94 ± 2.82	35.76 ± 1.91
	PRE (%)	92.73 ± 0.01	79.43 ± 3.15	95.00 ± 0.84	80.97 ± 5.37	95.14 ± 0.53	95.64 ± 0.39	90.51 ± 1.77	89.92 ± 1.72
	SEN (%)	92.83 ± 0.76	77.37 ± 3.99	95.45 ± 0.52	81.85 ± 1.79	94.27 ± 1.30	95.14 ± 1.31	90.68 ± 1.87	89.66 ± 1.65
	SPC (%)	99.05 ± 0.08	99.12 ± 0.16	99.30 ± 0.11	99.91 ± 0.02	99.95 ± 0.01	99.92 ± 0.01	99.96 ± 0.01	99.60 ± 0.06

## Data Availability

The fetal data used to support the findings of this study were provided by the University Children’s Hospital Zurich and can be available at https://doi.org/10.7303/syn23747212 (accessed on 5 September 2021). The dataset is cited at relevant places within the text as reference [18].
